# The efficacy and safety of Huangqi Guizhi Wuwu decoction for rheumatoid arthritis: A protocol for systematic review and meta-analysis

**DOI:** 10.1097/MD.0000000000022011

**Published:** 2020-09-04

**Authors:** Lei Wang, Yihua Fan, Ping Xin, Yuetong Zhao, Huaihan Deng, Bo Jia

**Affiliations:** a Chengdu University of Traditional Chinese Medicine, Chengdu, Sichuan Province; b First Teaching Hospital of Tianjin University of Traditional Chinese Medicine; c Tianjin University of Traditional Chinese Medicine, Tianjin; d Pengzhou Hospital of Traditional Chinese Medicine, Pengzhou, Sichuan Province, China.

**Keywords:** Huangqi Guizhi Wuwu decoction, protocol, randomized controlled trials, rheumatoid arthritis, systematic evaluation

## Abstract

**Background:**

Rheumatoid arthritis has the characteristics of slow progression, long course, and repeated attacks. At present, western medicine commonly used in clinical practice not only reduces pain and improves symptoms, but also has more adverse reactions, affecting the health, and life of patients. In ancient China, Huangqi Guizhi Wuwu decoction was used by doctors to treat rheumatoid arthritis, with remarkable effect. In recent years, many clinical studies have also shown that Huangqi Guizhi Wuwu decoction has reliable effect in treating rheumatoid arthritis, but there is no evidence of evidence-based medicine. Therefore, this study aims to systematically evaluate the clinical efficacy and safety of Huangqi Guizhi Wuwu decoction in the treatment of rheumatoid arthritis.

**Methods:**

Using computer to retrieve PubMed, The Cochrance Library, Embase, Web of Science, CNKI, VIP and Wanfang database, in addition manually retrieve Google academic and Baidu academic to collect all randomized controlled trials for Huangqi Guizhi Wuwu decoction in the treatment of rheumatoid arthritis, including relevant academic journal and conference papers, dissertations, from the establishment of the database to July 2020. After 2 evaluators independently screened the literature, extracted the data, and evaluated the risk of bias included in the study, RevMan5.3 software was used to analyze the data.

**Results:**

This research evaluate the efficacy and safety of Huangqi Guizhi Wuwu decoction in treating Rheumatoid arthritis from the aspects of clinical efficacy rate, visual analog scale (VAS), swollen joint count (SJC), morning stiffness time, Rrythrocyte sedimentation rate (ESR), C-reactive protein (CRP), rheumatoid factor (RF), and adverse reaction incidence.

**Conclusion:**

This study will provide reliable evidence for the clinical application of Huangqi Guizhi Wuwu decoction in the treatment of rheumatoid arthritis.

**Ethics and dissemination:**

The private information from individuals will not publish. This systematic review also will not involve endangering participant rights. Ethical approval is not required. The results may be published in a peer-reviewed journal or disseminated in relevant conferences.

**OSF Registration number:**

DOI 10.17605/OSF.IO/RZY3V.

## 1 Introduction

Rheumatoid arthritis (RA) is an autoimmune disease, also is 1 of common diseases in clinical, with the main clinical manifestations of symmetry, aggressive little joint swelling and pain, often involving multiple joints, and gradually appears bone tissue damage, has a high rate of disability, seriously affects patients daily life.^[1–3]^ More than 6 million people in China suffer from rheumatoid arthritis, with an incidence of around 0.34%.^[4]^ The pathogenesis of rheumatoid arthritis is still unclear, and non-steroidal anti-inflammatory drugs, glucocorticoids, antirheumatic drugs, and biological agents are commonly used in clinical practice to relieve patients pain, delay the destruction of joint tissues and protect other organs from damage.^[5,6]^

As long-term oral western medicine has relatively large side effects and single effect, it often needs to be combined with other medicines,^[7]^ resulting in poor compliance of patients and relatively difficult treatment.

Huangqi Guizhi Wuwu decoction is a classic prescription in ancient China, which can be used for diabetic peripheral neuropathy,^[8]^ cervical spondylosis of nerve root type^[9]^ and rheumatoid arthritis,^[10]^ etc. As a result, the prescription has effects of invigorating qi, blood, and warming the meridians,^[11]^ and it also has good curative effect in anti-inflammation analgesic, improving immunity and so on.

Although there are many clinical studies have shown that Huangqi Guizhi Wuwu decoction has the significant effect of RA, and also has certain curative effect combined western medicine therapy, However, there are differences in the research plan and evaluation of curative effect among clinical trials, which lead to the uneven results, the lack of evidence, to a certain extent affect the reliability of the results of the study and promotion of the therapy. Therefore, this study collected randomized controlled trials of Huangqi Guizhi Wuwu decoction combined with western medicine in the treatment of RA. According to the systematic evaluation method of Cochrane cooperation network, the effectiveness, and safety of Huangqi Guizhi Wuwu decoction in the treatment of rheumatoid arthritis were evaluated objectively, to provide reliable evidence-based basis for clinical application of Huangqi Guizhi Wuwu decoction in RA.

## 2 Methods

### 2.1 Protocol register

This protocol of systematic review and meta-analysis has been drafted under the guidance of the preferred reporting items for systematic reviews and meta-analyses (PRISMA). Moreover, it has been registered on open science framework (OSF) on July 29, 2020 (Registration number: DOI 10.17605/OSF.IO/RZY3V).

### 2.2 Ethics

Since this is a protocol with no patient recruitment and personal information collection, the approval of the ethics committee is not required.

### 2.3 Eligibility criteria

We will collected all available randomized controlled trails (RCTs) on Huangqi Guizhi Wuwu decoction treatment for RA, regardless of blinding, publication status, region, but Language will be restricted to Chinese and English.

#### 2.3.1 Research subjects

Patients who meet the diagnostic criteria of rheumatoid arthritis, among which the nationality, race, age, sex, course of disease, the location of the disease is unlimited.

#### 2.3.2 Intervention measures

The control group was treated with western medicine alone, and the types, dosage, and course of treatment of western medicine were not limited; the treatment group was treated with Huangqi Guizhi Wuwu decoction, including Huangqi Guizhi Wuwu decoction, or Huangqi Guizhi Wuwu decoction combined with western medicine, Among them, Huangqi Guizhi Wuwu decoction type (granule or decoction), dosage, medication methods were not limited.

#### 2.3.3 Outcome indicators

1. Primary outcome: the overall effective rate;
2. Secondary outcomes: ①Visual analog scale (VAS); ②Swollen joint count (SJC); ③Morning stiffness time; ④C-reactive protein (CRP); ⑤Rrythrocyte sedimentation rate (ESR); ⑥Rheumatoid factor (RF); ⑦Incidence of adverse reactions.

### 2.4 Exclusion criteria

1. As for repeatedly published study, choose the 1 with the most complete data;
2. The article that publishes as abstract or study whose data is incomplete, and relevant literature cannot be obtained after contacting the author;
3. Studies with obvious data errors;
4. The treatment group was the study of Huangqi Guizhi Wuwu decoction combined with other TCM therapies, such as combined with other TCM compounds, acupuncture, or massage.
5. Literature with high bias risk assessed by randomization or allocation concealment.^[12]^

### 2.5 Search strategy

CNKI, Wanfang Data, VIP, PubMed, Cochrane Library, Embase, Web of Science, etc. were retrieved by computer from the establishment of the database to July 2020, with the retrieval words “Rheumatoid Arthritis ”,“Huangqi Guizhi Wuwu Decoction ”, “Rheumatoid ”, etc, in Chinese and “arthritis, rheumatoid ”, “rheumatoid arthritis ”, “ostarthritis ”, “Huangqi Guizhi Wuwu Decoction ”, etc, in English, and both use a combination of subject words and free words. In addition, manually retrieve in Baidu academic, Google academic to collect all domestic and foreign literatures about Huangqi Guizhi Wuwu decoction in the treatment of rheumatoid arthritis. Taking PubMed as an example, the search strategy is shown in Table 1.

**Table 1:**
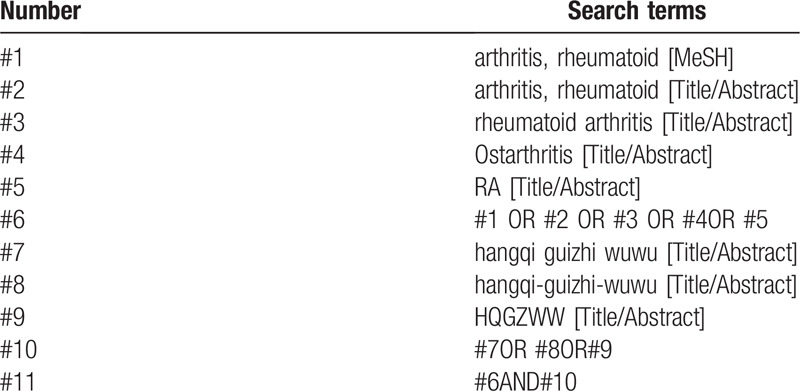
Search strategy in PubMed database.

### 2.6 Data screening and extraction

**Figure 1:**
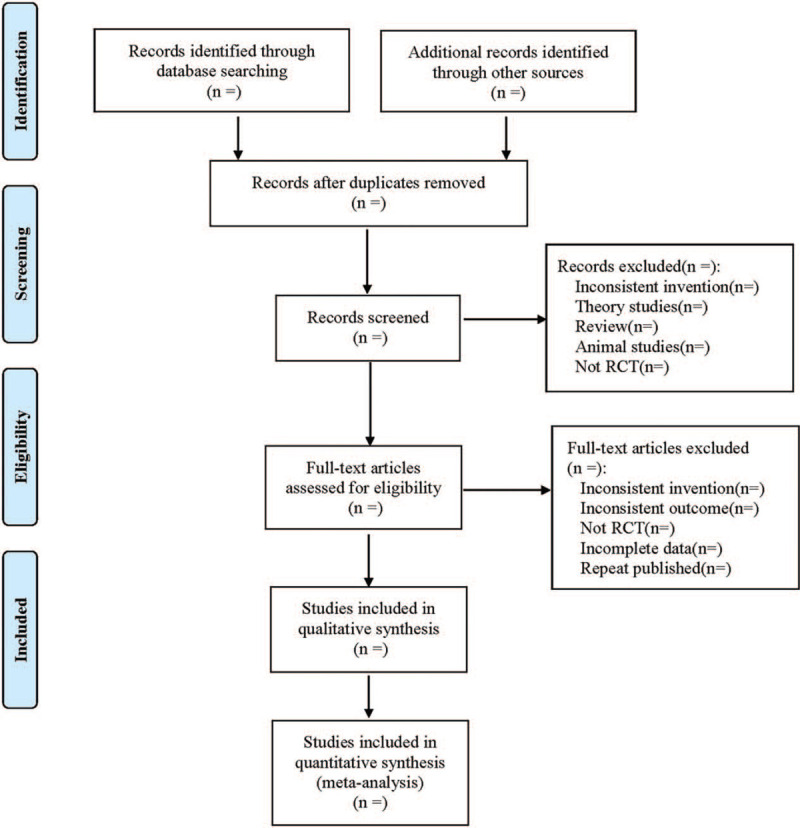
Flow diagram.

Cochrane Collaboration System Reviewer Manual Version 5.0 was used as a reference for the method of selection in the study. According to the PRISMA flow chart, EndNote X^7^ document management software was utilized by 2 researchers to independently screen the documents based on the above inclusion and exclusion criteria, duplicate and obviously unqualified studies were removed, and the remaining literature was searched for original text for further screening. During the second screening, the 2 researchers read the literature independently to determine whether they met the inclusion criteria, and then the 2 exchanged the results of the inclusion test, for studies where there was disagreement and it was difficult to determine whether to include or not, a third investigator discussed the decision. At the same time, Excel 2013 was used to extract relevant information, including: ① Clinical research (title, first author, publication year, and month, sample size, sex ratio, average age, average course of disease); ② Intervention measures (name, dosage, course of treatment of western medicine used in the control group; the dosage form, dosage, and course of treatment of Huangqi Guizhi Wuwu decoction or usage and dosage of other western medicine used in the treatment group); ③ Evaluation factors of risk bias in randomized controlled studies; ④ Observation indicators. The literature screening process is shown in Figure 1.

### 2.7 Literature quality evaluation

Using the Cochrane collaborations tool for assessing risk of bias to assess the risk of bias of the included study. According to the performance of the included literature in the above evaluation items, the 2 researchers will give low risk, unclear, and high risk judgments one by one, and cross-check after completion respectively. In case of any disagreement, discussion will be carried out. If no agreement can be reached, discussion will be made with the researchers in the third party.

### 2.8 Statistical analysis

#### 2.8.1 Data analysis and processing

The RevMan 5.3 software was used for statistical analysis. For dichotomous variables, relative risk (RR) was used for statistics. For continuous outcomes, weighted mean difference (WMD) was selected when the tools and units of measurement indicators are the same, standardized mean difference (SMD) was selected when the tools and units of measurement indicators are different, and all the above were represented by effect value and 95% confidence interval (CI). The heterogeneity was determined by *χ*^*2*^ and *I*^*2*^ values. If (*P* ≥ . 1, *I*^*2*^ ≤ 50%) indicated low heterogeneity, fixed effect model was used for meta-analysis. If (*P <* . 1, *I*^*2*^ > 50%) indicated heterogeneity among studies, and the source of heterogeneity would be explored through subgroup analysis. If there was no obvious clinical or methodological heterogeneity, it would be considered as statistical heterogeneity, and the random-effect model would be used for analysis. Descriptive analysis was used instead of meta-analysis if there was significant clinical heterogeneity between the 2 groups and subgroup analysis was not available.

#### 2.8.2 Dealing with missing data

If there is missing data in the article, contact the author via email for additional information. If the author cannot be contacted, or the author has lost relevant data, descriptive analysis will be conducted instead of meta-analysis.

#### 2.8.3 Subgroup analysis

Do respectively meta-analysis according to the treatment group of Huangqi Guizhi Wuwu decoction alone and Huangqi Guizhi Wuwu decoction combined with Western medicine; subgroup analysis was carried out according to different dosage forms of Huangqi Guizhi Wuwu decoction; subgroup analysis was conducted according to the course of treatment of Chinese herbal compound; subgroup analysis was carried out according to different western medicine in the control group.

#### 2.8.4 Sensitivity analysis

In order to determine the stability of outcome indicators, sensitivity analyze was used to analyze each outcome indicator.

#### 2.8.5 Assessment of reporting biases

Funnel plots were used to assess publication bias if no fewer than 10 studies were included in an outcome measure. Moreover, Eggers and Beggs test were used for the evaluation of potential publication bias.

#### 2.8.6 Evidence quality evaluation

The Grading of Recommendations Assessment, Development, and Evaluation (GRADE) will be used to assess the quality of evidence. It contains 5 domains (bias risk, consistency, directness, precision, and publication bias). And the quality of evidence will be rated as high, moderate, low, and very low.

## 3 Discussion

RA is an immunological disease mainly characterized by synovitis of the joints, and is highly prevalent in people aged 30 to 50 years.^[13]^ RA is a chronic disease. Long-term use of western medicine may cause adverse reactions such as vomiting, nausea, and liver, and kidney function injury, bringing great pain to patients.^[14]^ Through the treatment of syndrome differentiation, the effect of traditional Chinese medicine in the treatment of rheumatoid arthritis is significant, and for its less adverse reactions, many patients recognize it.

RA belongs to the category of “Arthralgia syndrome”(Bizheng) in Chinese medicine. It was mentioned in *“Synopsis of the Golden Chamber”(Jingui Yaolue)* in the Eastern Han Dynasty that the pathogenic factors of wind, cold, dampness, and heat invade the patient with deficiency of the whole body from the outside, with the passage of time, causing the deficiency of zhengqi and fullness of pathogenic factors, leading to the decline of the functions of the zang-fu organs, poor circulation of qi and blood, obstruction of the meridians, resulting in swelling, pain and stiffness of the joints, and thus the onset of arthrosis. Huangqi Guizhi Wuwu decoction, also from *“Synopsis of the Golden Chamber”(Jingui Yaolue)*, is composed of Huangqi (*Radix Astragali*), Guizhi (*Ramulus Cinnamomi*), Baishao (*Raidix Paeoniae Alba*), Shengjiang (*Rhizoma Zingiberis Recens*), Dazao (*Fructus Jujubae*), and has beneficial effects of tonifying qi and blood, warming the meridians, and tongbi.^[15]^ Huangqi (*Radix Astragali*) in the prescription is the king medicine, has the beneficial effect of benefiting qi and strengthening superficies, inducing diuresis to alleviate edema; the pungent-warm Guizhi (*Ramulus Cinnamomi*) can dredge the meridians, help Yangqi; Baishao (*Raidix Paeoniae Alba*) can harmonize yingxue, and combined with Guizhi (*Ramulus Cinnamomi*) can harmonize ying and wei, nourish blood and tongbi; Shengjiang (*Rhizoma Zingiberis Recens*) can not only dispel pathological wind outside, but also help Yangqi and xuan bi; Dazao (*Fructus Jujubae*) harmonizes all medicines. The whole prescription plays the effects of invigorating qi and dispelling pathogenic factors, relieving pain through arteries and veins, which is suitable for the basic pathogenesis of RA based on qi deficiency and meridians obstruction.^[16]^

Huangqi (*Radix Astragali*) can increase the number of cells in cell culture, reduce platelet adhesion, and enhance immune and anti-inflammatory functions.^[17,18]^ Guizhi (*Ramulus Cinnamomi*) and Shengjiang (*Rhizoma Zingiberis Recens*) also have anti-inflammatory and analgesic effects.^[19]^ Studies have found that^[20,21]^ Huangqi Guizhi Wuwu decoction can effectively relieve the symptoms of RA patients, such as joint swelling, pain, morning stiffness, etc., and reduce ESR, CRP, and other laboratory indicators, thus improving the patients quality of life.

Through this study, the efficacy and safety of Huangqi Guizhi Wuwu decoction in the treatment of rheumatoid arthritis can be systematically evaluated, and the difference in efficacy between simple Huangqi Guizhi Wuwu decoction and western medicine treatment can be obtained, as well as whether the efficacy of Huangqi Guizhi Wuwu decoction combined with western medicine is superior to that of western medicine alone. In addition, the adverse reactions of Huangqi Guizhi Wuwu decoction can be learned, which is conducive to the promotion of clinical use. However, this study also has some limitations, including few literatures and small sample size. In addition, since the treatment group adopted Huangqi Guizhi Wuwu decoction plus and less treatment, rather than Huangqi Guizhi Wuwu decoction original prescription, the medication and dose were different, and there was certain heterogeneity. In addition, this study only searched English and Chinese literature, and may ignore studies or reports in other languages, with certain publication bias. Therefore, high-quality and large sample literature support is still needed in order to improve the reliable basis for clinicians and patients to use Huangqi Guizhi Wuwu decoction to treat RA.

## Author contributions

**Data collection:** Ping Xin and Yuetong Zhao.

**Funding to support:** Bo Jia.

**Literature search:** Lei Wang and Yihua Fan.

**Software operation:** Huaihan Deng.

**Supervision:** Bo Jia.

**Writing – original draft:** Lei Wang and Yihua Fan.

**Writing – review & editing:** Lei Wang and Bo Jia.
